# The Relationships and Mediating Effects of Work–Life Factors on Acute and Chronic Fatigue Among Nurses

**DOI:** 10.1155/jonm/6087500

**Published:** 2026-09-21

**Authors:** Chia Jung Hu, Feng Ping Lee, Rei Mei Hong

**Affiliations:** ^1^ School of Nursing, Kaohsiung Medical University, Kaohsiung City, Taiwan, kmu.edu.tw; ^2^ Department of Medical Research, Kaohsiung Medical University Chung-Ho Memorial Hospital, Kaohsiung City, Taiwan, kmu.edu.tw; ^3^ School of Health Sciences, University of the Pacific, Sacramento, USA, pacific.edu; ^4^ Department of Nursing, Chang Gung University of Science and Technology, Chiayi Campus, Chiayi County, Taiwan, cgust.edu.tw

**Keywords:** fatigue, nurse, occupational health, work–life style

## Abstract

**Background:**

Fatigue is a prevalent occupational health concern among nurses. Inadequate recovery from acute fatigue may contribute to persistent fatigue, while work‐related and life‐related factors may influence this process.

**Aim:**

To examine factors associated with chronic fatigue and the mediating roles of life‐related factors in the association between acute and chronic fatigue among Taiwanese nurses.

**Methods:**

A cross‐sectional study was conducted. A total of 322 clinical nurses were recruited through online forums and social media groups and completed a web‐based questionnaire assessing sociodemographic characteristics, work‐related factors, health‐promoting lifestyle behaviors, and occupational fatigue. Analysis of variance, Pearson’s correlation, hierarchical multiple regression, and partial least squares structural equation modeling with bootstrapping were used.

**Findings:**

Acute fatigue was strongly correlated with chronic fatigue (*r* = 0.70), whereas intershift recovery was negatively correlated with chronic fatigue (*r* = −0.58). The addition of intershift recovery significantly increased the explained variance in chronic fatigue by 2.0% beyond acute fatigue and work‐related factors. Life‐related factors further increased the explained variance by 2.6%, while intershift recovery remained negatively associated with chronic fatigue in the adjusted model (*β* = −0.19, *p* < 0.001). Mediation analysis further showed significant indirect associations through interpersonal support and stress management, whereas the indirect effect through physical activity was not significant.

**Conclusions:**

Acute fatigue is strongly associated with chronic fatigue, while adequate intershift recovery and psychosocial and lifestyle‐related factors may be important in limiting fatigue accumulation.

**Implications for Nursing Management:**

Nurse managers should support adequate recovery between shifts, manage psychological work demands, and strengthen interpersonal support and stress‐management resources to reduce persistent fatigue and promote nurses’ long‐term well‐being.

## 1. Background

Coronavirus disease 2019 (COVID‐19), caused by severe acute respiratory syndrome coronavirus 2 (SARS‐CoV‐2), was declared a pandemic in 2020 [1]. According to official statistics, Taiwan ranked among the top 10 countries globally in terms of daily confirmed COVID‐19 cases per million population between May and June 2022 [2]. Rapid increases in the number of hospitalized COVID‐19 patients place healthcare professionals at risk of infection; this constant risk is a source of stress and fatigue [3].

A study found high levels of emotional exhaustion (70.9%) among critical healthcare professionals in Taiwan, regardless of whether they directly cared for COVID‐19 patients, particularly among younger, unmarried staff, longer hours, and night shifts [4]. Similarly, a survey of American nurses also showed that, in the later stages of the pandemic, nurses remained at risk; two‐thirds of acute care nurses reported feeling stressed and exhausted [5]. Studies have found that fatigue has direct negative effects on nurses’ physical and mental health. Consequently, many nurses have chosen to leave the profession [6].

Fatigue is a complex physiological phenomenon caused by long waking hours, a high workload, poor health status, and a poor work–life balance [7]. It is generally categorized as acute fatigue or chronic fatigue based on its onset and duration. While acute fatigue typically resolves with adequate rest, insufficient recovery may cause fatigue to accumulate and eventually progress into chronic fatigue, with intershift recovery identified as a key mediating factor in preventing this transition among hospital nurses [8].

During the COVID‐19 pandemic, nurses faced significant limitations in taking vacation or restorative time off, reducing their ability to recover from workplace‐related fatigue. Consequently, it is crucial to investigate how life‐related factors may mitigate the progression of fatigue in this population. Prior studies have identified that shift‐working nurses can benefit from lifestyle‐based strategies, such as exercise and social interaction, to manage fatigue [9]. Notably, high‐frequency and long‐duration physical activity appears particularly effective in alleviating fatigue symptoms [10].

Life‐related factors—including social support, physical activity, and stress management, have been recognized as critical contributors to both psychological and physiological well‐being. According to the stress‐buffering model, social support—encompassing emotional and tangible forms—can reduce the adverse effects of stress [11]. Physical activity has also been shown to modulate immune function, lower proinflammatory cytokine levels, and stabilize circadian rhythms, thereby enhancing sleep and energy regulation [12]. Furthermore, mindfulness‐ and relaxation‐based stress‐management interventions have demonstrated effectiveness in reducing cortisol levels, improving stress resilience [13].

Although previous studies have highlighted the importance of life‐related factors such as social support, physical activity, and stress management in mitigating fatigue, few have clearly differentiated between acute and chronic fatigue or explored how these two states are developmentally linked. In particular, the role of life‐related factors in the association between acute and chronic fatigue remains underexplored. These relationships are especially important for shift‐working nurses who face ongoing recovery limitations, particularly during prolonged health crises such as the COVID‐19 pandemic. To address this gap, the present study examines whether key life‐related factors, namely interpersonal support, physical activity, and stress management, mediated the relationship between acute and chronic fatigue among nurses in Taiwan during the later stages of the pandemic. The following hypotheses are proposed:

 H1: Work–life‐related factors, acute fatigue, and intershift recovery are significantly associated with chronic fatigue.
 H2: Life‐related factors mediate the relationship between acute fatigue and chronic fatigue after controlling for work‐related variables.

## 2. Methods

### 2.1. Study Designs

Nurses working in teaching hospitals or medical centers with more than 1 year of work experience, in direct contact with or caring for patients, and understanding Mandarin were eligible for this cross‐sectional study. The exclusion criterion was OPD nurses. For SEM, a sample size of > 300 cases was suggested [14]. In total, 322 valid questionnaires were returned online, providing a reliable dataset.

### 2.2. Ethical Considerations

This study was a part of a health promotion survey among nurses, which was reviewed and approved by the Hospital Ethics Committee of JEN AI Hospital, Taiwan (IRB no. 111‐30). Considering the constraints posed by the COVID‐19 pandemic, the authors adopted a convenience sampling approach to recruit participants from internet groups of hospital nurses in Taiwan. The participants were encouraged to pass the web questionnaire link on to other eligible nurses. After the participants had read the online informed consent form and agreed to participate by ticking a checkbox, they completed the anonymous online questionnaire. The questionnaire was prepared using Google Forms. Each online account could complete the questionnaire only once. The study was conducted between July 1 and July 31, 2022, that is, after the peak period of COVID‐19 infections every day, about 30,0000 cases in Taiwan, in the “plateau period” [2]. The questionnaire covered participants’ sociodemographic characteristics, work–life factors, and acute and chronic fatigue.

### 2.3. Sociodemographic Characteristics

The sociodemographic characteristics of interest included gender (male/female), age group (10‐year intervals), marital status (single/married), education (associate’s/bachelor’s/master’s or above), and teaching hospital (regional/medical center).

### 2.4. Work‐Related Factors

The workstyle information of interest included shift work (day/afternoon/night), average daily working hours (8 ≤ *x* < 9 h, 9 ≤ *x* < 10 h, 10 ≤ *x* ≤ 12 h), and psychological work demands.

Psychological work demands (seven items) were measured using the subscales of the Job Content Questionnaire [15], which is based on a 4‐point Likert‐type scale (1 = *strongly disagree*, 4 = *strongly agree*). The questions were My job requires me to do things very quickly/My job requires me to be very hard‐working/I am not asked to do an excessive amount of work/I have enough time to get the job done/My job requires me to concentrate for a long time/My job is very busy/There is a shortage of staff in my workplace. Two items were reverse scored. Scores ranged from 7 to 28 points, with higher scores indicating greater psychological work demands [15]. The Cronbach’s *α* was 0.77.

### 2.5. Life Factors (Interpersonal Support, Physical Activity, and Stress Management)

These factors were obtained from the subscales of the Chinese Health‐Promotion Lifestyle Profile [16]. In accordance with [11–13], we used three potentially related subscales: interpersonal support (six items), physical activity (six items), and stress management (seven items), all of which were scored using 4‐point Likert‐type scales (1 = *never*, 2 = *sometimes*, 3 = *often*, and 4 = *routinely*). The total score was 6–24 for physical activity and interpersonal support and 7–28 for stress management, with higher scores indicating healthier lifestyles. Cronbach’s αs for interpersonal support, physical activity, and stress management were 0.91, 0.84, and 0.69, respectively.

### 2.6. Acute Fatigue, Intershift Recovery, and Chronic Fatigue

The Chinese Version of the Occupational Fatigue Exhaustion Recovery Scale (OFER) was used to measure acute fatigue, intershift recovery, and chronic fatigue [17]. The OFER consists of three subscales—acute fatigue, intershift recovery, and chronic fatigue—with each subscale comprising five items rated on a 7‐point Likert‐type scale (0 = *strongly disagree*, 6 = *strongly agree*). The scores were calculated as follows: [sum (scale scores)/(*n* items in scale × 6)] × 100. Scores ranged from 0 to 100 points. Higher acute and chronic fatigue scores indicate greater fatigue, whereas higher intershift recovery scores indicate better recovery between work shifts. According to the scoring interpretation of the Chinese OFER, scores of 1–25 indicate a low level, 26–50 a low‐to‐moderate level, 51–75 a moderate‐to‐high level, and 76–100 a high level of the respective construct. Thus, for the intershift recovery subscale, higher scores represent greater recovery from work‐related fatigue before the subsequent shift. Example items for acute fatigue included: “After a typical work shift, I have little energy left” and “I usually feel exhausted when I come home from work.” For chronic fatigue, sample items included: “I often wonder how long I can keep going in my work” and “I feel at the end of my rope with my work” [17]. The intershift recovery subscale was used to assess the extent to which participants recovered from work‐related fatigue during the nonwork period between consecutive shifts. Cronbach’s *α* was 0.81 for acute fatigue, 0.79 for intershift recovery, and 0.86 for chronic fatigue.

### 2.7. Statistical Analysis

The relationships between the factors of interest, acute fatigue, intershift recovery, and chronic fatigue were analyzed using descriptive statistics, analysis of variance (ANOVA), Pearson’s correlation analysis, and hierarchical multiple regression in SPSS Statistics 24 (IBM Corp., Armonk, NY, USA). To examine the mediating effects of life‐related factors on the association between acute and chronic fatigue, path analysis was conducted using Smart PLS 4 (Smart PLS GmbH, Schleswig‐Holstein, Germany), with 5000 bootstrap samples for robust estimation. Following the recommendations [14], this model was designed with an emphasis on parsimony and theoretical clarity. Therefore, other variables not directly related to the main hypotheses were excluded to maintain the model’s conceptual focus and estimation efficiency.

Prior to conducting SEM, we assessed the normality of the data. Skewness and kurtosis values fell within the acceptable range (−1 to 1), confirming normality. Additionally, the composite reliability and factor loadings of each latent variable were evaluated. Cronbach’s *α* remained above 0.6, and all factor loadings exceeded 0.5 [14]. Statistical significance was set at *p* < 0.05.

## 3. Results

The study included 322 clinical nurses, predominantly female (96.3%), with a bachelor’s degree (76.4%), and employed in local or teaching hospitals (72.0%). Most were aged 21–50 years, and 62.7% worked afternoon or night shifts. Acute fatigue differed significantly by shift type and average daily working hours. Nurses working afternoon/night shifts reported higher acute fatigue than those working day shifts, and those working 9–12 h per day reported higher acute fatigue than those working 8–9 h per day. Chronic fatigue differed significantly by daily working hours, with higher scores among nurses working 9–12 h than among those working 8–9 h. No significant differences in acute or chronic fatigue were observed according to gender, marital status, age, education level, or hospital type (Table 1).

**TABLE 1:** Fatigue scores according to demographic and workstyle characteristics (*N* = 322).

	*N*	%	Acute fatigue score				Chronic fatigue score			
			M	SD	*t*/*F*	*p*	M	SD	*t*/*F*	*p*
Gender					2.82	0.094			0.14	0.708
a). Male	12	3.7	59.72	20.52			74.72	19.51		
b). Female	310	96.3	67.53	15.59			72.67	18.52		
Marital status					0.00	0.983			2.60	0.108
a). Single	150	46.6	67.26	17.00			74.53	17.83		
b). Married	172	53.4	67.22	14.79			71.20	19.03		
Age (y)					0.71	0.545			0.68	0.563
a). 21–30	85	26.4	66.50	16.04			71.29	19.22		
b). 31–40	102	31.7	68.69	16.03			74.54	16.69		
c). 41–50	109	33.9	67.24	16.01			72.87	19.76		
d). 51–60	26	8.1	63.97	13.72			70.00	18.16		
Education					1.39	0.24			1.52	0.220
a). Associate degree	32	9.9	69.37	14.91			74.58	15.35		
b). Bachelor’s degree	246	76.4	67.58	15.47			73.30	18.27		
c). Master’s degree and above	44	13.7	63.78	18.20			68.33	21.60		
Hospital type					0.09	0.761			1.64	0.201
a). Local/teaching hospital	232	72.0	67.41	15.80			73.57	18.78		
b). Medical center	90	28.0	66.81	16.02			70.62	17.79		
Shift work					4.28	0.039^∗^			2.84	0.093
a). Day shift	120	37.3	64.88	15.69		b > a	70.50	19.31		
b). Afternoon/night shift	202	62.7	68.64	15.79			74.09	17.96		
Average daily working hours					15.95	0.000^∗∗∗^			11.03	0.000^∗∗∗^
a). 8 ≤ *x* < 9 h	94	29.2	59.82	17.09		b > a	65.42	18.96		b > a
b). 9 ≤ *x* < 10 h	210	65.2	70.36	13.96		c > a	75.73	17.61		c > a
c). 10≤ *x* ≤ 12 h	18	5.6	69.62	17.55			76.29	16.87		

^∗^ *p* < 0.05.

^∗∗∗^ *p* < 0.001.

The mean scores for acute fatigue, chronic fatigue, and intershift recovery were 67.24 (SD = 15.83), 72.75 (SD = 18.53), and 37.39 (SD = 16.14), respectively. Acute fatigue was positively correlated with chronic fatigue, whereas intershift recovery was negatively correlated with both acute and chronic fatigue. Chronic fatigue was also positively associated with psychological work demands and negatively associated with interpersonal support, physical activity, and stress management (Table 2).

**TABLE 2:** Pearson correlations between study variables (*N* = 322).

Items	M	SD	1	2	3	4	5	6	7
1. Chronic fatigue	72.75	18.53	1						
2. Acute fatigue	67.24	15.83	0.70^∗∗∗^	1					
3. Intershift recovery	37.39	16.14	−0.58^∗∗∗^	−0.64^∗∗∗^	1				
4. Psychological work demands	22.61	2.88	0.43^∗∗∗^	0.47^∗∗∗^	−0.41^∗∗∗^	1			
5. Interpersonal support	17.10	3.69	−0.32^∗∗∗^	−0.36^∗∗∗^	0.20^∗∗∗^	−0.06	1		
6. Physical activity	11.26	3.49	−0.21^∗∗^	−0.35^∗∗∗^	0.56^∗∗∗^	−0.07	0.39^∗∗∗^	1	
7. Stress management	21.25	4.46	−0.50^∗∗∗^	−0.46^∗∗∗^	0.17^∗∗^	−0.10	0.67^∗∗∗^	0.37^∗∗∗^	1

^∗∗^ *p* < 0.01.

^∗∗∗^ *p* < 0.001.

Hierarchical multiple regression showed that acute fatigue and work‐related factors explained 51.5% of the variance in chronic fatigue in Model 1. Adding intershift recovery significantly increased the explained variance by 2.0%, with better intershift recovery associated with lower chronic fatigue. The addition of health‐promoting lifestyle factors further increased the explained variance by 2.6%. The final model explained 56.1% of the variance in chronic fatigue. Acute fatigue remained the strongest factor associated with chronic fatigue (*β* = 0.508, *p* < 0.001), while intershift recovery (*β* = −0.193, *p* < 0.001), psychological work demands, physical activity, and stress management were also significant. Interpersonal support and working‐hour categories were not significant in the final model (Table 3).

**TABLE 3:** Hierarchical multiple regression analysis of factors associated with chronic fatigue (*N* = 322).

Predictor	Model 1					Model 2					Model 3				
	*B*	SE	*β*	95% CI for *B*	*p*	*B*	SE	*β*	95% CI for *B*	*p*	*B*	SE	*β*	95% CI for *B*	*p*
Acute fatigue	0.753	0.05	0.64	0.65 to 0.86	< 0.001	0.63	0.06	0.54	0.50 to 0.75	**< 0.001**	0.60	0.07	0.5	0.47 to 0.72	**< 0.001**

Working hours															
(a). 8 ≤ *x* < 9 h	(ref)					(ref)					(ref)				
(b). 9 ≤ *x* < 10 h	0.94	1.71	0.02	−2.42 to 4.32	0.581	0.88	1.68	0.02	−2.42 to 4.19	0.599	1.21	1.65	0.03	−2.03 to 4.46	0.463
(c). 10 ≤ *x* ≤ 12 h	1.67	3.41	0.02	−5.03 to 8.369	0.624	2.09	3.34	0.03	−4.48 to 8.664	0.532	4.32	3.34	0.05	−2.24 to 10.90	0.196

Psychological work demands	0.79	0.29	0.12	0.22 to 1.36	**0.007**	0.61	0.29	0.10	0.05 to 1.18	**0.035**	0.69	0.29	0.11	0.12 to 1.25	**0.017**

Intershift recovery						−0.22	0.06	−0.19	−0.33 to −0.10	**< 0.001**	−0.22	0.06	−0.19	−0.33 to −0.11	**< 0.001**

Interpersonal support											−0.39	0.24	−0.08	−0.86 to 0.09	0.108

Physical activity											0.66	0.24	0.12	0.18 to 1.13	**0.007**

Stress management											−0.75	0.27	−0.13	−1.28 to −0.22	**0.005**

	Adjusted *R* ^2^ = 0.509 *F* = 84.16^∗∗∗^					Adjusted *R* ^2^ = 0.528 *F* = 72.77^∗∗∗^ Δ*R* ^2^ = 0.020 Δ*F* = 13.71^∗∗∗^					Adjusted *R* ^2^ = 0.550 *F* = 49.96^∗∗∗^ Δ*R* ^2^ = 0.026 Δ*F* = 6.09^∗∗∗^				

*Note:* ^∗^< 0.05, ^∗∗^< 0.01, ^∗∗∗^< 0.001.

**FIGURE 1:**
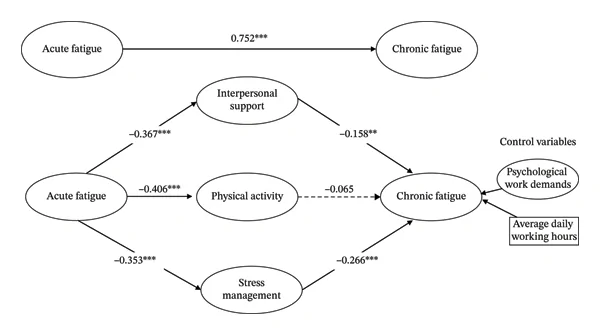
Mediation model of the association between acute and chronic fatigue through interpersonal support, physical activity, and stress management. Note. Values represent path coefficients. ^∗∗^ *p* < 0.01; ^∗∗∗^ *p* < 0.001.

The PLS–SEM results showed a significant direct association between acute and chronic fatigue (path coefficient = 0.752, *p* < 0.001). Significant indirect effects were observed through interpersonal support (indirect effect = 0.06, *p* = 0.023) and stress management (indirect effect = 0.09, *p* = 0.003), whereas the indirect effect through physical activity was not significant (indirect effect = 0.02, *p* = 0.377) (Figure 1 and Table 4).

**TABLE 4:** Factors mediating the transition from acute to chronic fatigue: results of path analysis (*N* = 322).

Direct effects	Original sample (O)	Sample mean (M)	STDEV	*T*	5000 bootstraps bias‐corrected 95% CI		*p*
					Lower	Upper	
Acute fatigue ⟶ chronic fatigue	0.75	0.75	0.02	31.68	0.70	0.80	< 0.001^∗∗∗^
Interpersonal support ⟶ chronic fatigue	−0.16	−0.16	0.06	2.76	−0.28	−0.05	0.006^∗∗^
Physical activity ⟶ chronic fatigue	−0.07	−0.07	0.07	0.97	−0.19	0.07	0.331
Stress management ⟶ chronic fatigue	−0.27	−0.28	0.06	4.23	−0.37	−0.12	< 0.001^∗∗∗^
Specific indirect effects							
Acute fatigue ⟶ interpersonal support ⟶ chronic fatigue	0.06	0.06	0.02	2.27	0.00	0.11	0.023^∗^
Acute fatigue ⟶ physical activity ⟶ chronic fatigue	0.02	0.03	0.03	0.63	−0.03	0.08	0.377
Acute fatigue ⟶ stress management⟶ chronic fatigue	0.09	0.10	0.03	3.02	0.02	0.14	0.003^∗∗^
Total indirect effects							
Acute fatigue⟶ chronic fatigue	0.17	0.18	0.03	4.61	0.09	0.23	< 0.001^∗∗∗^

*Note:* STDEV, standard deviation; Original sample: effect value.

Abbreviation: CI, confidence interval.

^∗^ *p* < 0.05.

^∗∗^ *p* < 0.01.

^∗∗∗^ *p* < 0.001.

## 4. Discussion

This study demonstrates that chronic fatigue is significantly associated with acute fatigue, intershift recovery, psychological work demands, physical activity, and stress management. Acute fatigue remained the strongest factor associated with chronic fatigue, while better intershift recovery was independently associated with lower chronic fatigue. Mediation analysis further showed significant indirect effects through interpersonal support and stress management, whereas the indirect effect through physical activity was not significant. These findings highlight the importance of both work‐related demands and recovery‐related factors in chronic fatigue among nurses.

Previous studies have identified work experience and shift work as significant predictors of fatigue [8, 18, 19]. While work experience typically shows a negative association with fatigue, and night shifts have been linked to higher fatigue levels, our findings did not reveal significant differences in chronic fatigue by age, gender, marital status, hospital type, or shift type. This is consistent with evidence from the COVID‐19 pandemic [20], suggesting that elevated stress levels during public health emergencies may override the protective effects of individual demographic factors. Although chronic fatigue differed according to daily working hours in the unadjusted analysis, working‐hour categories were no longer significant in the fully adjusted model. In contrast, psychological job demands remained positively associated with chronic fatigue, underscoring the continued impact of workload‐related stressors.

These findings suggest that prolonged working hours should be carefully managed, particularly in high‐stress environments, while ensuring sufficient recovery time between shifts. Supporting this recommendation, a previous study found that without appropriate interventions, emergency medical services clinicians working two consecutive 12‐h shifts required at least three consecutive days of rest to recover fully [21]. In the present study, better intershift recovery remained independently associated with lower chronic fatigue even after controlling for acute fatigue and other related factors. Additionally, we observed a medium positive association between psychological job demands and chronic fatigue, consistent with earlier research [22]. This highlights the need for organizational strategies aimed at managing mental workload, especially during periods of sustained crisis such as the COVID‐19 pandemic.

Beyond occupational and sociodemographic factors, life‐related aspects also play an important role in fatigue development and recovery. Interpersonal support was negatively correlated with chronic fatigue in the bivariate analysis, although it was not independently associated with chronic fatigue in the fully adjusted model. Stress management, however, remained significantly associated with lower chronic fatigue. These factors may contribute to psychological well‐being and facilitate recovery from work‐related stress. According to the acute fatigue reservoir model [23], adequate rest is essential for restoring the physical and psychological resources required to meet occupational demands. Similarly, previous research has emphasized that maintaining a healthy lifestyle can mitigate emotional exhaustion and improve overall well‐being [24].

Although interpersonal support was not statistically significant in the fully adjusted model, it may still provide emotional and instrumental resources that help reduce perceived stress and bolster resilience against work‐related fatigue. Prior research suggests that women are more likely than men to rely on interpersonal support as a coping strategy for regulating emotions and managing workplace stress [11, 25]. Given the predominantly female composition of our sample, social support networks may therefore remain relevant to fatigue and occupational stress in this population.

In addition to interpersonal support, evidence indicates that individuals working long hours or with irregular and unpredictable schedules tend to experience reduced control over their personal lives. These conditions are commonly associated with sleep disturbances, reduced physical activity, and unhealthy lifestyle habits, such as poor diet [24, 26]. Chronic exposure to such stressors without appropriate coping mechanisms may lead to cumulative fatigue and burnout. Conversely, structured stress management strategies—such as mindfulness, relaxation training, and cognitive‐behavioral approaches—have been shown to reduce psychological strain, restore a sense of control, and buffer against fatigue accumulation. Furthermore, cultivating meaningful relationships has been linked to enhanced self‐esteem, increased motivation, and greater engagement in health‐promoting behaviors [11, 27]. These findings underscore the importance of workplace wellness programs that integrate psychosocial support and stress management interventions to mitigate long‐term fatigue risk.

Physical activity and exercise play an important role in regulating circadian rhythms and improving sleep quality, thereby helping to reduce fatigue caused by insufficient rest. Moderate‐intensity exercise interventions lasting at least 6 weeks are beneficial for fatigue and energy [10]. However, in this study, physical activity did not significantly mediate the link between acute and chronic fatigue. The nonsignificant indirect effect of physical activity may reflect differences in the frequency, intensity, or type of physical activity among participants. This is consistent with other evidence showing that overall physical activity levels among nurses are generally low to moderate [28, 29]. Even though day‐shift nurses tend to have higher step counts—around 8700 steps per day—their total physical activity still falls below international recommendations [28]. These findings suggest that future workplace health programs should encourage nurses to engage in regular, structured moderate‐to‐vigorous physical activity to help reduce long‐term fatigue risks.

Despite these limitations, this study provides an integrated perspective by examining work‐related factors, intershift recovery, and health‐promoting lifestyle factors in relation to chronic fatigue. In particular, the findings highlight acute fatigue as the strongest factor associated with chronic fatigue and suggest that adequate recovery between shifts and effective stress management may be important considerations for workforce planning and well‐being strategies, particularly in prolonged high‐stress environments such as pandemics.

### 4.1. Implications for Nursing Management

Nurse managers should prioritize adequate recovery between shifts and manage psychological work demands to reduce the accumulation of fatigue. Organizational strategies should also strengthen interpersonal support and provide accessible stress‐management resources. Integrating recovery‐supportive scheduling, peer support, and stress‐management interventions into workplace wellness programs may help reduce persistent fatigue and promote nurses’ long‐term well‐being.

## 5. Conclusions

This study highlights the pivotal role of acute fatigue as the factor most strongly associated with chronic fatigue, particularly among nurses working under prolonged crisis conditions, such as the COVID‐19 pandemic. Importantly, better intershift recovery was independently associated with lower chronic fatigue, suggesting that adequate recovery between shifts may be important in limiting the accumulation of fatigue. Recognizing this pathway, our findings highlight that interpersonal support and stress management are essential in interrupting this progression from acute fatigue to longer‐term fatigue states. Fatigue is influenced not only by physical workload but also by psychosocial and lifestyle‐related factors that affect recovery from acute fatigue episodes. Strengthening protective life factors—such as personal support and effective stress management—can help against the accumulation of acute fatigue and reduce the risk of persistent fatigue. To address this, healthcare institutions should implement integrated wellness programs that include accessible stress management training and peer support systems. Nevertheless, long working hours and insufficient recovery time remain primary contributors to fatigue becoming persistent, as they exacerbate unresolved acute fatigue. These structural issues must be addressed alongside individual‐level interventions. Future research should adopt longitudinal approaches to evaluate how effectively such interventions disrupt the acute‐to‐chronic fatigue trajectory and how organizational policies and working conditions shape this process, ultimately supporting nurse well‐being and long‐term workforce sustainability.

## Author Contributions

Conceptualization: Chia Jung Hu and Rei Mei Hong; methodology: Chia Jung Hu, Feng Ping Lee, and Rei Mei Hong; data analysis: Chia Jung Hu and Feng Ping Lee; writing–original draft preparation: Chia Jung Hu; writing–review and editing: Chia Jung Hu, Feng Ping Lee, and Rei Mei Hong.

## Funding

The authors received no specific funding for this work.

## Ethics Statement

This study was approved by the Hospital Ethics Committee of JEN AI Hospital, Taiwan (IRB no. 111‐30). Informed consent was obtained from all participants. All methods were carried out in accordance with relevant guidelines and regulations.

## Consent

The authors have nothing to report.

## Conflicts of Interest

The authors declare no conflicts of interest.

## Data Availability

The data that support the findings of this study are available from the corresponding author upon reasonable request.
